# Endoscopic and clinicopathological characteristics of colorectal T/NK cell lymphoma

**DOI:** 10.1186/s13000-020-01044-5

**Published:** 2020-10-21

**Authors:** Hideki Ishibashi, Satoshi Nimura, Fumihito Hirai, Naohiko Harada, Hiromi Iwasaki, Sigeto Kawauchi, Yumi Oshiro, Atsuji Matsuyama, Shotaro Nakamura, Yasushi Takamatsu, Hirotoshi Yonemasu, Taturo Shimokama, Morishige Takeshita

**Affiliations:** 1 Department of Gastroenterology and Medicine, Faculty of Medicine, Fukuoka University, 7-45-1 Nanakuma, Johnan-ku, Fukuoka, Fukuoka 814-0180 Japan; 2 Department of Pathology, Fukuoka University, 7-45-1 Nanakuma, Johnan-ku, Fukuoka, Fukuoka 814-0180 Japan; 3 Department of Gastroenterology National Hospital Organization, Kyushu Medical Center, 1-8-1 Jigyohama, Chuo-ku, Fukuoka, Fukuoka 810-8563 Japan; 4 Department of Hematology National Hospital Organization, Kyushu Medical Center, 1-8-1 Jigyohama, Chuo-ku, Fukuoka, Fukuoka 810-8563 Japan; 5 Department of Pathology, National Hospital Organization, Kyushu Medical Center, 1-8-1 Jigyohama, Chuo-ku, Fukuoka, Fukuoka 810-8563 Japan; 6 Division of Pathology, Matsuyama Red Cross Hospital, 1 Bunkyocho, Matsuyama, Ehime 790-8524 Japan; 7 Department of Pathology and Oncology, School of Medicine, University of Occupational and Environmental Health, 1-1 Yahatanishi-ku, Kitakyushu, Fukuoka, 807-8555 Japan; 8 Division of Gastroenterology, Iwate Medical University, 2-1-1 Shiwa-gun, Morioka, Iwate 020-8505 Japan; 9 Division of Medical Oncology, Hematology and Infectious Diseases, Faculty of Medicine, Fukuoka University, 7-45-1 Nanakuma, Johnan-ku, Fukuoka, Fukuoka 814-0180 Japan; 10 Department of Pathology, Oita Red Cross Hospital, 3-2-37 Chiyomachi Oita, Oita, 870-0033 Japan; 11 Department of Pathology, Steel Memorial Yahata Hospital, 1-1-1 Yahatahigashi-ku, Kitakyushu, Fukuoka, 805-8508 Japan

**Keywords:** Colorectum, MEITL, ATLL, T/NK-cell lymphoma, Lymphocytic proctocolitis

## Abstract

**Background:**

Colorectal T/natural killer (NK)-cell lymphomas (TNKCL) are very rare. Endoscopic and clinicopathological characteristics of colorectal TNKCL have not been clearly demonstrated. In this study, we demonstrated the clinical characteristics of colorectal TNKCL.

**Methods:**

Endoscopic and clinicopathological characteristics were investigated in 27 patients with colorectal monomorphic epitheliotropic intestinal T-cell lymphoma (MEITL), adult T-cell leukemia/lymphoma (ATLL), and other types of TNKCL.

**Results:**

Nine TNKCL patients (33%) were classified as MEITL, 11 (41%) as ATLL, and seven (26%) as other. Four patients with Epstein-Barr Virus-positive (EBV+) TNKCL, two indolent T-cell lymphoproliferative disorder and one anaplastic large cell lymphoma were included in the other group. Endoscopically, six MEITL (67%) and five ATLL (46%) showed diffuse-infiltrating type, in which the main endoscopic lesion was edematous mucosa in MEITL, while aphthoid erosion and edematous mucosa were typical in ATLL. Ulcerative type was identified in four other group patients (57%), including two EBV+ TNKCL. An increase in atypical T-intraepithelial lymphocytes (T-IELs) was noted in seven MEITL (88%) and six ATLL (60%) patients, but not in the other group (0%) patients. Five MEITL patients (56%) showed features of lymphocytic proctocolitis with increased CD8+ T-IELs.

**Conclusions:**

MEITL and ATLL occasionally invaded the colorectum, and primary involving MEITL was observed. Diffuse infiltrating type was the characteristic endoscopic finding in colorectal MEITL and ATLL, while ulcerative type was observed in the other group. Features of lymphocytic proctocolitis may be prodromal findings of MEITL.

## Background

The gastrointestinal (GI) tract is the most involved site of extranodal non-Hodgkin’s lymphoma, and the stomach and small intestine are the most affected organs [1, 2]. Primary GI tract lymphomas are mainly composed of B-cell neoplasia, which involve the whole GI tract, including the colorectum [3]. T/natural killer (NK)-cell lymphoma (TNKCL) of the GI tract has occasionally been reported [4, 5]. Enteropathy-associated T-cell lymphoma (EATL) frequently occurs in Europe and the United States, and consists of CD3+, CD30+/negative, CD56 negative lymphoma cells, which are closely associated with celiac disease [6]. Monomorphic epitheliotropic intestinal T-cell lymphoma (MEITL) mainly shows medium-sized CD8+, CD56+/negative lymphoma cells, and is more prevalent in East Asia, having no correlation with celiac disease [7]. Intestinal Epstein-Barr virus-positive (EBV+) TNKCL is mainly reported in East Asia and consists of CD56+/negative and CD8+/negative lymphoid cells [8, 9]. Indolent T-cell lymphoproliferative disorder (TLPD) of the GI tract mainly comprises non-epitheliotropic CD4+ small lymphoid cells [10]. EATL, MEITL, EBV+ TNKCL and indolent TLPD mainly involve the small intestine, and colorectal tumor invasion has occasionally been reported [6–12]. Adult T-cell leukemia/lymphoma (ATLL) consists of CD4+ T-cell neoplasm and frequently presents leukemic changes and tumor cell invasion in various organs, including the GI tract [13, 14]. Primary colorectal B-cell lymphoma is found mainly in the ileocecum and endoscopically shows predominantly ulcerative and polypoid types, while diffuse infiltrating type is rare [15]. Small and large intestinal TNKCL including colorectal MEITL and EBV+ TNKCL often presents ulcero-infiltrative, ulcerating, and ulcerofungating type lesions [16]. However, the endoscopic and clinicopathological characteristics of colorectal TNKCL have rarely been reported worldwide [16, 17].

The current study reported 27 patients with colorectal TNKCL, with MEITL and ATLL patients accounting for more than two-thirds of the patient population. Detailed endoscopic and clinicopathological characteristics were demonstrated in the TNKCL patients. Furthermore, features of lymphocytic proctocolitis was detected in MEITL patients. The relationship between lymphocytic proctocolitis and TNKCL was discussed.

## Methods

### Patient selection and clinical findings

Institutional ethical approval was obtained in compliance with the Declaration of Helsinki (institutional review board approval number: U20–06-006). We retrospectively retrieved records and samples of TNKCL of the GI tract from 106 patients (32 MEITL, 61 ATLL, 13 other types of TNKCL) taken between 1990 and 2018 at the Department of Pathology, Fukuoka University. Histological classification was performed according to the World Health Organization (WHO) classification and reference of intestinal EBV+ TNKCL in 2017 [6–8, 10]. The current study focused on 27 Japanese patients with colorectal TNKCL. Clinical stage was determined according to the Lugano classification [18].

### Endoscopic examination

**Fig. 1:**
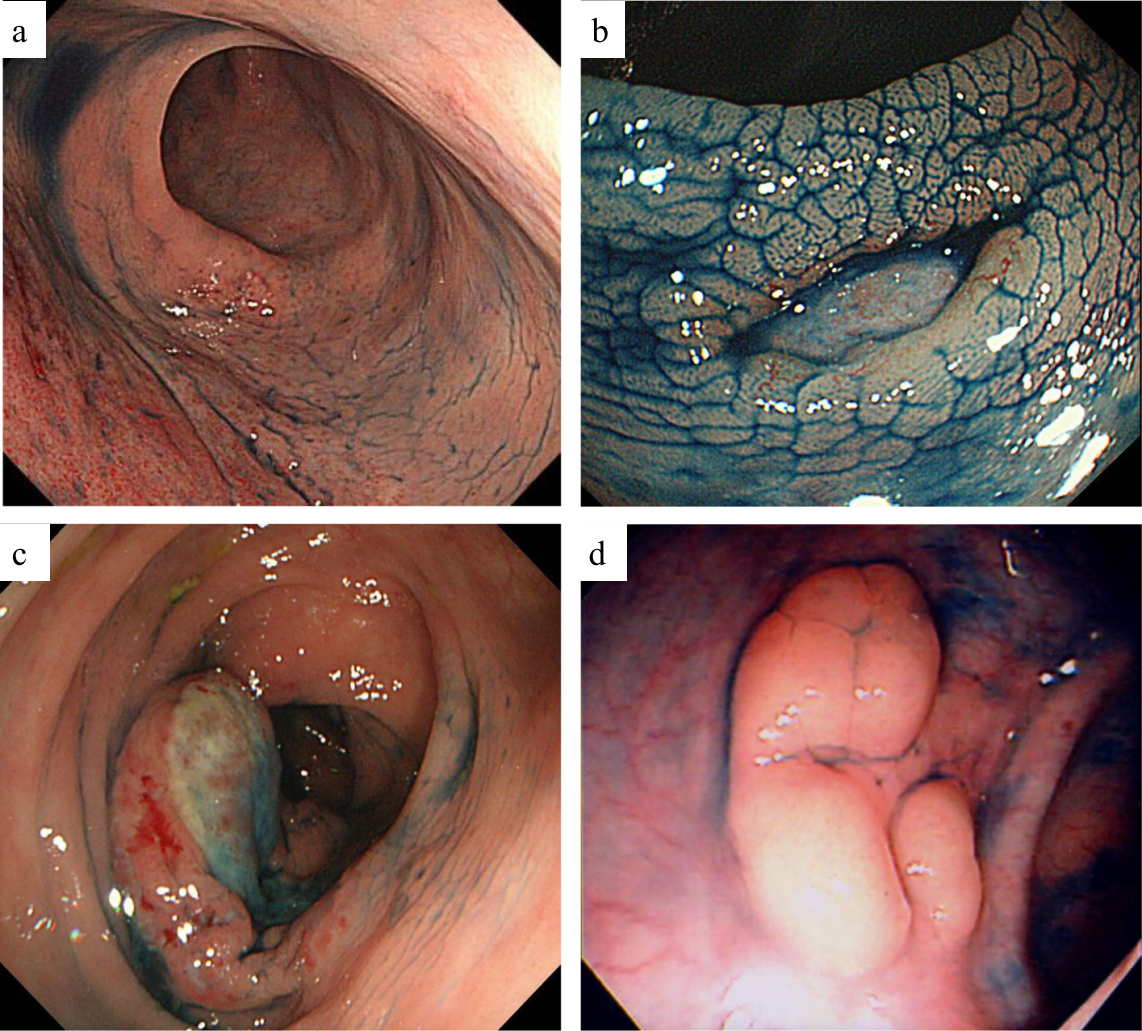
Endoscopic findings of patients with colorectal TNKCL. Low GI endoscopic views of the colon showing (**a**) diffuse infiltrating type MEITL, ulcerative type (**b**) ATLL and (**c**) EBV+ CD56 negative TNKCL, and (**d**) polypoid type ATLL

All 27 patients underwent endoscopic examination of the upper and/or lower GI tract. A video endoscope (Olympus Medical System, Tokyo, Japan) was used for all endoscopic examinations. The endoscopic images or records were reviewed retrospectively by experienced endoscopists (H.I, F.H). Biopsy specimens were taken from the stomach and/or small intestine and/or colon by standard biopsy forceps for histologic examination. Endoscopic findings of the main lesions of colorectal TNKCL were classified into three types based on a previous classification [19]: 1) diffuse infiltrating (colitis-like or proctocolitis-like lesion); 2) ulcerative (including stricturing and non-stricturing); and 3) polypoid (localized) (Fig. 1).

### Histology and immunohistochemistry

Histologic examination was performed in biopsy specimens of the GI tract from all 27 TNKCL patients and in colorectal surgical specimens from seven. In addition to the cytohistological characteristics of tumors, two additional histologic findings were examined due to similar features between MEITL and ATLL [20]. Medium-sized and large atypical lymphocytes with irregular nuclei infiltrating the epithelium were defined as atypical intraepithelial lymphocytes (IELs). In benign-looking mucosal layers, more than 20 small IELs per 100 epithelial cells were considered as findings of lymphocytic proctocolitis. For immunohistology, monoclonal and polyclonal antibodies were applied to formalin-fixed tumor samples using a Leica Bond-III automated stainer (Leica Biosystems, Buffalo Grove, IL). Immunostaining of CD3 (PS1; Leica Biosystems, Newcastle, UK), CD4 (4B12; Leica Biosystems), CD8 (C81/44B, Leica Biosystems), CD25 (4C9; Leica Biosystems), CD56 (1B6, Leica), CD30 (BerH2, DakoCytomation, Glostrup, Denmark), CD194 (chemokine receptor (CCR) 4, 1G1; BD Bioscience, San Jose, CA), CD103 (EPR4166^2^; Abcam, Cambridge, MA), and TIA1 (2GP; Immunotech, Marseille, France) was performed after antigen retrieval. Samples in which more than 30% of the tumor cells were labeled with a particular antibody marker were classified as positive. Pathological findings were reviewed by two pathologists (S.N and M.T).

### Statistical analysis

Clinicopathological data were analyzed using the chi-squared test and Fisher’s exact test. A *P*-value of < 0.05 was considered statistically significant. Overall survival (OS) curves of all examined patients were generated using the Kaplan–Meier method and analyzed by the log-rank test.

## Results

### Patient characteristics and treatments

The clinical features of 27 patients with colorectal TNKCL are summarized in Table 1. Nine patients (33%) were classified as MEITL, 11 (41%) as ATLL, and seven (26%) as other (one EBV+ CD56+; three EBV+ CD56 negative TNKCL; two indolent TLPD; one anaplastic large cell lymphoma (ALCL)). The median patient age at diagnosis was 60 years (range, 43–84 years). One patient with MEITL and one with EBV+ CD56+ TNKCL each had a long history of ulcerative colitis (UC) treated with mesalazine, of 13 and 17 years, respectively. The most frequently encountered clinical symptom was chronic diarrhea in five MEITL (56%) and four ATLL (36%) patients. The levels of serum lactate dehydrogenase (LDH) and soluble interleukin-2 receptor (sIL-2R) in MEITL patients were significantly lower than those of ATLL patients (*P* < 0.01). Leukemic change was frequently found in six of eight ATLL patients (75%) compared with two of nine MEITL (22%) (*P* < 0.05). Two MEITL patients (22%) and two of the other group (EBV+ CD56 negative TNKCL and indolent TLPD) (29%) were classified as stage I. One MEITL (11%) and one other group patient (indolent TLPD) (14%) were classified as stage II1. Six patients were considered as primary colorectal TNKCL without other GI tumor involvement. Four MEITL patients (44%), 10 ATLL (91%), and three other group patients (two EBV+ TNKCL and one ALCL) (42%) were classified as stage IV. Four MEITL (44%), one ATLL (9%), and two other group (both EBV+ CD56 negative TNKCL) (29%) patients underwent colectomy to remove the main tumors. Three MEITL (33%) and one ATLL (9%) patients received chemotherapy after surgery. Fourteen patients (52%) received combined chemotherapy. Four MEITL and seven ATLL patients received the CHOP regimen (cyclophosphamide, doxorubicin, vincristine, and prednisolone). Three MEITL patients underwent the CHASE (cyclophosphamide, cytarabine, etoposide, and dexamethasone) and SMILE (methotrexate, ifosfamide, etoposide, l-asparaginase, and dexamethasone) regimens. Mogamulizumab was additionally administered to two ATLL patients. Three patients (11%) received stem cell transplantation after chemotherapy, and three patients (11%) were not treated.

**Table 1:** Clinical findings and treatments of 27 colorectal T/NK cell lymphoma patients

Clinical subtype	MEITL	ATLL	Other group	Total
No. of cases	9 (33%)	11 (41%)	7 (26%)	27
Median age (range), y	63 (47–84)	64 (50–74)	54 (43–69)	60 (43–84)
Male: female	6:3	7:4	6:1	19:8
Past history of UC	1 (11%)	0	1 (14%)	2 (7%)
Chronic diarrhea	5 (56%)	4 (36%)	2 (29%)	11 (41%)
Abdominal pain	4 (44%)	3 (27%)	4 (57%)	11 (41%)
Weight loss	4 (44%)	2 (18%)	0	6 (22%)
Fever	0	2 (18%)	2 (29%)	4 (15%)
Total protein (g/dl)	5.7 (4–6.6)	6.3 (5.4–7.2)	7.5 (5.9–8.4)	6.5 (4–8.4)
Albumin (g/dl)	3.1 (2.1–3.8)	3.4 (2.6–4)	3.9 (2.7–4.4)	3.5 (2.1–4.4)
LDH (U/l)	191 (112–288)^*^	785 (262–2192)^*^	184 (123–244)	387 (112–2192)
sIL-2R (U/ml)	1815 (587–4410)^*^	51,643 (3501–154,246)^*^	10,993 (1330–25,818)	21,484 (587–154,246)
Leukemic change	2 (22%)^**^	6/8 (75%)^**^	1 (14%)	9/21 (43%)
Clinical stage I	2 (22%)	0	2 (29%)	4 (15%)
II (II1, II2, IIE)	3 (33%)	1 (9%)	2 (29%)	6 (22%)
II1	1 (11%)	0	1 (14%)	2 (7%)
II2	1 (11%)	0	0	1 (4%)
IIE	1 (11%)	1 (9%)	1 (14%)	3 (11%)
IV	4 (44%)^**^	10 (91%)^**^	3 (42%)	17 (63%)
Surgery	1 (11%)	0	2 (29%)	3 (11%)
Surgery and chemotherapy	3 (33%)	1 (9%)	0	4 (15%)
Chemotherapy	2 (22%)^**^	9 (82%)^**^	3 (42%)	14 (52%)
Chemotherapy and SCT	2 (22%)	1 (9%)	0	3 (11%)
No treatment	1 (11%)	0	2 (29%)	3 (11%)

^*^ *P* < 0.01; ^**^ *P* < 0.05; *UC* Ulcerative colitis; *LDH* Lactate dehydrogenase; *sIL-2R* Soluble interleukin-2 receptor

SCT, stem cell transplantation

### Involved GI sites and endoscopic findings of colorectal TNKCL

**Fig. 2:**
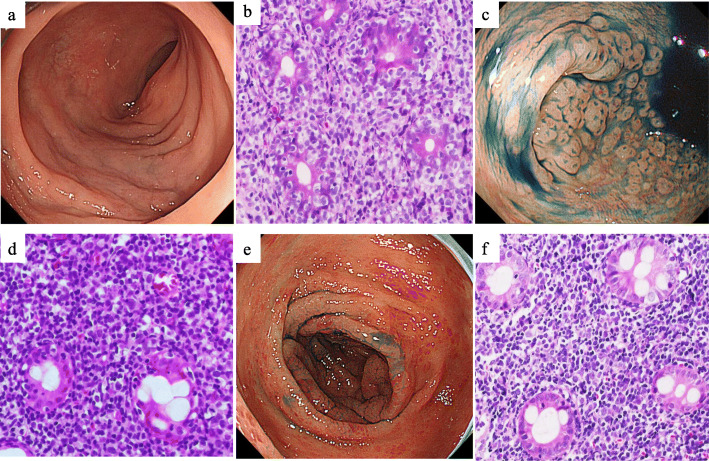
Endoscopic and histologic findings of diffusely infiltrating type MEITL (a, b) and ATLL (c-f). **a** Endoscopic view of sigmoid colon showing mild edematous mucosa with small erosion. **b** Histological analysis showing small nests of atypical intraepithelial lymphocytes (IELs), preserved glands, and invasion by medium-sized atypical lymphocytes (hematoxylin and eosin stain, × 400). **c** Endoscopic view of cecum showing many aphthoid colitis-like lesions. **d** Histological analysis showing preserved glands, small nests of atypical IELs, and invasion by pleomorphic medium-sized atypical lymphocytes, × 400. **e** Endoscopic view of the descending colon showing reddish, mildly edematous mucosa. **f** Histological analysis showing invasion by small and medium-sized atypical lymphocytes, × 400

Involved GI sites and endoscopic findings of the main lesions in 27 colorectal TNKCL patients are summarized in Table 2. Three MEITL (33%), seven ATLL (64%), and five other group (four EBV+ TNKCL and one indolent TLPD) (71%) patients showed colorectal invasion without tumor invasion into the stomach and small intestine. The small intestine and duodenum were the other GI-involved sites of lymphoma in six MEITL patients, and the small intestine, duodenum, and stomach were the involved sites of four ATLL. Endoscopically, six MEITL (67%), five ATLL (46%), and two other group (EBV+ CD56+ TNKCL and indolent TLPD) (29%) patients showed diffuse infiltrating type lesions, among which four MEITL patients showed edematous mucosa (Fig. 2a, b), and three and two ATLL patients showed mainly aphthoid erosion (Fig. 2c, d) and edematous mucosa (Fig. 2e, f), respectively. Four patients of the other group (two EBV+ CD56 negative TNKCL, one indolent TLPD and one ALCL) (57%), one MEITL (11%), and three ATLL (27%) patients showed ulcerative type lesions. Two MEITL (22%) and three ATLL (27%) patients showed polypoid type lesions. Seven MEITL patients (78%), seven ATLL (64%), and one other group (EBV+ CD56+ TNKCL, 14%) showed more than two colorectal lesions by lymphoma cells. Four MEITL patients (44%), five ATLL (46%), and one of the other group (EBV+ CD56+ TNKCL, 14%) showed multiple invasions by lymphoma cells from the cecum to the rectum.

**Table 2:** Involved gastrointestinal sites and endoscopic findings in 27 colorectal T/NK cell lymphoma patients

	MEITL (*n* = 9)	ATLL (*n* = 11)	Other group (*n* = 7)	Total (*n* = 27)
Gastrointestinal sites				
Colon	3 (33%)	7 (64%)	5 (71%)	15 (56%)
Colon+other	6 (67%)	4 (36%)	2 (29%)	12 (44%)
Colon+small intestine	2 (22%)	1 (9%)	0	3 (11%)
Colon+small intestine+duodenum	4 (44%)	0	0	4 (15%)
Colon+duodenum+stomach	0	2 (18%)	1 (14%)	3 (11%)
Colon+stomach	0	1 (9%)	1 (14%)	2 (7%)
Endoscopic findings				
Diffuse-infiltrating	6 (67%)	5 (46%)	2 (29%)	13 (48%)
Ulcerative	1 (11%)	3 (27%)	4 (57%)	8 (30%)
Polypoid	2 (22%)	3 (27%)	1 (14%)	6 (22%)
Involved colon sites				
C/A/T/D/S/R	5/6/5/4/6/6	6/7/8/6/6/6	1/4/2/1/2/2	12/17/15/11/14/14
Localized lesion	2 (22%)	4 (36%)	6 (86%)	12 (44%)
Multiple lesions	7 (78%)	7 (64%)	1 (14%)	15 (56%)
Two lesions	3 (33%)	1 (9%)	0	4 (15%)
Three lesions (T + D + R)	0	1 (9%)	0	1 (4%)
Six lesions (C + A + T + D + S + R)	4 (44%)	5 (46%)	1 (14%)	10 (37%)

*C* Cecum; *A* Ascending colon; *T* Transverse colon; *D* Descending colon; *S* Sigmoid colon; *R* Rectum

### Pathological and immunohistological findings of colorectal TNKCL

**Fig. 3:**
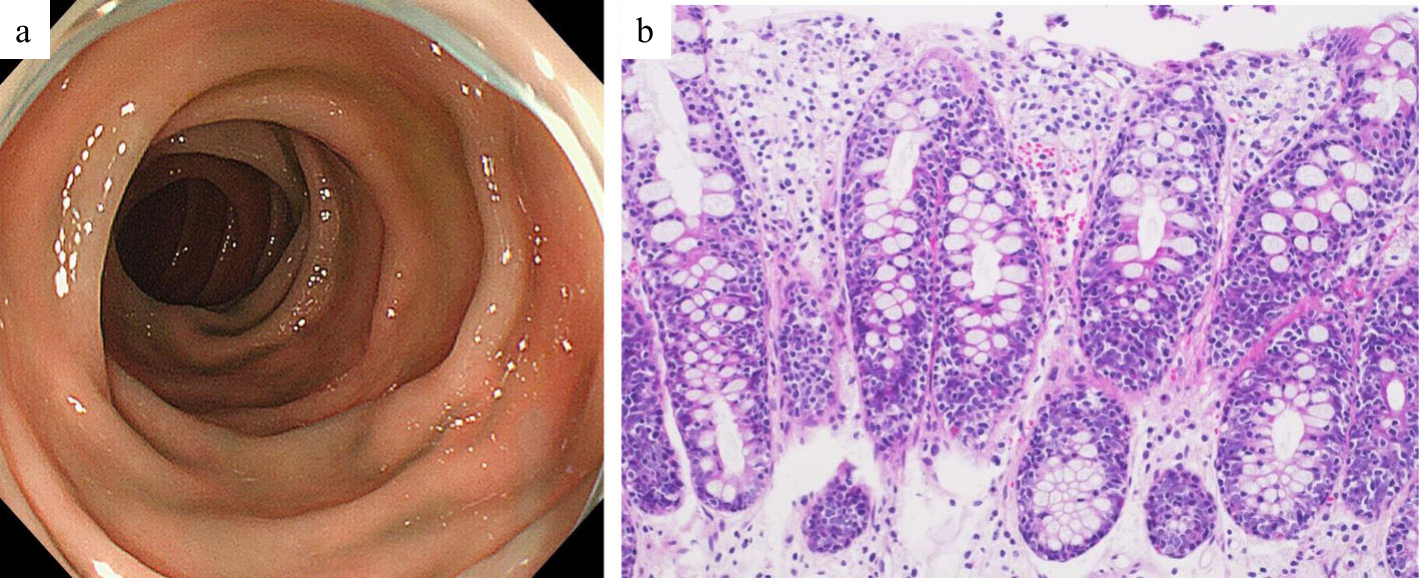
Lower GI endoscopic and histologic findings of features of lymphocytic colitis in MEITL. **a** Endoscopic view of the descending colon showing mild edematous mucosa. **b** Histological analysis showing many scattered small IELs and no overt stromal invasion by lymphoma cells (hematoxylin and eosin stain, × 200)

The pathological and immunohistological findings of 27 colorectal TNKCL patients are summarized in Table 3. Histologically, all nine MEITL patients (100%) had monomorphic medium-sized cell lymphoma, while eight ATLL patients (73%) had pleomorphic medium-sized cell lymphoma. In the other group, one EBV+ CD56+ TNKCL and two indolent TLPD (42%) were composed of pleomorphic medium-sized cell lymphoma, and three EBV+ CD56 negative TNKCL and one ALCL (57%) showed monomorphic large cell lymphoma. Seven of eight MEITL patients (88%) and six of 10 ATLL (60%) showed an increase in atypical IELs in the overlying epithelium and in glands in and near the tumors. These findings were not observed in other group patients (0%). Five MEITL patients (56%) showed features of lymphocytic proctocolitis, in which an increase in reactive small IELs was found in the background nonneoplastic mucosa (Fig. 3a, b), and one ATLL (9%) showed an increase in reactive small IELs. Immunohistologically, lymphoma cells in all nine MEITL patients were CD3+, CD8+, and TIA-1+, and eight (89%) were CD56+. Atypical and reactive IELs were CD3+, CD8+, TIA-1+, and CD103+/−. Lymphoma cells in all 11 ATLL patients were CD3+, CD25+, and of those, nine (100%) were CD194+ (CCR4) and nine (82%) were CD4+. Six MEITL patients (67%), four ATLL (36%), and one other group patient (14%) showed CD103+ lymphoma cells. No EBERs+ tumor cells were found in MEITL and ATLL patients. In the other group, one patient showed nasal type EBV+ CD56+ TNKCL, and a further three had EBV+ CD56 negative and CD8+/negative lymphoma. Two indolent TLPD patients demonstrated CD4+ T-cells in the tumor, and one ALCL patient had CD30+, CD4+ and TIA1+ tumor cells.

**Table 3:** Pathological and immunohistological findings of 27 colorectal T/NK cell lymphoma patients

	MEITL (*n* = 9)	ATLL (*n* = 11)	Other group (*n* = 7)	Total (*n* = 27)
Histological findings				
Monomorphic medium-sized cell	9 (100%)^*^	1 (9%)^*^	0 (0%)	10 (37%)
Pleomorphic medium-sized cell	0 (0%)^*^	8 (73%)^*^	3 (42%)	11 (41%)
Monomorphic large cell	0 (0%)	0 (0%)	4 (57%)	4 (15%)
Pleomorphic large cell	0 (0%)	2 (18%)	0 (0%)	2 (7%)
Increased atypical IELs in tumors	7/8 (88%)	6/10 (60%)	0/5 (0%)	13/23 (57%)
Lymphocytic proctocolitis	5 (56%)^**^	1 (9%)^**^	0 (0%)	6 (22%)
Tumor cell markers				
CD3	9 (100%)	11 (100%)	5 (71%)	25 (93%)
CD4	1 (11%)^*^	9 (82%)^*^	3 (42%)	13 (48%)
CD8	9 (100%)^*^	2 (18%)^*^	2 (29%)	13 (48%)
CD25	0/8 (0%)^*^	11 (100%)^*^	2/6 (33%)	13/25 (52%)
CD56	8 (89%)^*^	1 (9%)^*^	1 (14%)	10 (37%)
CD30	0/8 (0%)	2/9 (22%)	4 (57%)	6/24 (25%)
CD194 (CCR4)	0/7 (0%)^*^	9/9 (100%)^*^	0 (0%)	9/23 (39%)
CD103	6 (67%)	4 (36%)	1 (14%)	11 (41%)
TIA-1	9 (100%)^*^	0/8 (0%)^*^	6 (86%)	15/24 (63%)
EBERs	0 (0%)	0 (0%)	4 (57%)	4 (15%)

^*^ *P* < 0.01; ^**^ *P* < 0.05; *IELs* Intraepithelial lymphocytes

### Analysis of patient prognosis

**Fig. 4:**
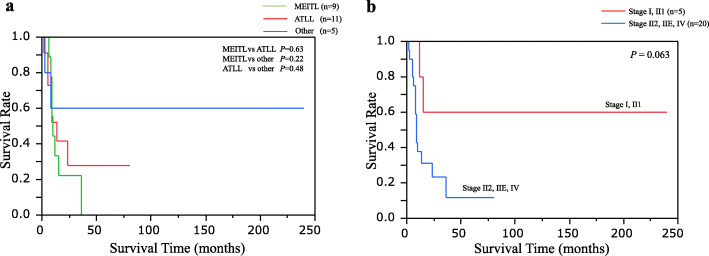
Overall survival (OS) curves in 25 patients with colorectal TNKCL. **a** 50% OS rates of MEITL, ATLL and other group were 9.5 months, 9 months, and more than 240 months, respectively. There was no statistical difference between MEITL and ATLL. **b** The 50% OS of five patients with primary colorectal TNKCL in early stages (I or II1) was over 240 months, and that of 20 patients in advanced stages was 8.5 months. There was no statistical difference between the two groups (*P* = 0.063)

The 50% OS of nine MEITL patients was 9.5 months, that of 11 ATLL was 9 months, and that of five other group patients was over 240 months. There was no prognostic difference in OS between MEITL and ATLL patients (Fig. 4a). The 50% OS of the five patients (three MEITL, one EBV+ CD56 negative TNKCL and one indolent TLPD) in the early stages (I and II1) was more than 240 months. The 50% OS of 20 patients in the advanced stages (II2, IIE and IV) was 8.5 months. There was no significant difference between the early and advanced stages (*P* = 0.063) (Fig. 4b).

## Discussion

Previous studies have shown that endoscopically, eight of 15 MEITL lesions (53.3%) and eight of 10 EBV+ TNKCL (80%) showed the ulcero-infiltrative type in 42 GI TNKCL [16]. Infiltrating and superficial/erosive type lesions were rarely found in each of the above histological groups. Another study demonstrated that nine intestinal EBV+ CD56+/negative TNKCL patients showed ulcerative (*n* = 5) and protruding (*n* = 4) type lesions [8]. Furthermore, 34 of 47 EBV+ CD56+ TNKCL patients (72.3%) frequently showed multiple ulcerative lesions [21]. In the current study, the ulcerative type was frequently encountered in four of seven other group patients (57%), in which two EBV+ CD56 negative TNKCL were included. Six MEITL patients (67%) and five ATLL (46%) mainly showed the diffuse infiltrating type. Although ulcerating and polypoid type lesions were also found in resting MEITL and ATLL patients, the diffuse infiltrating type was a frequently encountered characteristic of colorectal MEITL and ATLL, which was different from that of EBV+ TNKCL as well as B-cell lymphoma [3, 8, 16, 21]. Further detailed studies are necessary to clarify the endoscopic difference among MEITL, ATLL and EBV+ TNKCL.

Small intestinal MEITL mostly showed intramucosal spreading of lymphoma cells with neoplastic CD103+ and CD8+ T-IELs [11]. It was reported that lymphoma cells in 21 of 31MEITL (68%) as well as 31 of 56 GI ATLL patients (55%) showed expression of CD103 homing receptor of T-IELs, and ATLL had similar histopathological characteristics to MEITL with respect to increased atypical and reactive T-IELs [20]. The endoscopic findings of MEITL have been reported as edematous mucosa with mosaic and diffuse mucosal thickening patterns, and shallow ulceration, as well as ulcerative type tumors in the small intestine [22]. The endoscopic findings of GI-involved acute type ATLL showed superficial spreading-type lesions (equal to the diffuse infiltrating type) in 12 of 23 lesions (52%) [23]. In the current study, more than two colorectal lymphomatous lesions were frequently found in MEITL (78%) and ATLL (64%) patients. Histologically, increased atypical T-IELs were found in MEITL (88%) and ATLL (60%) patients. Although MEITL and GI ATLL showed distinct disease entities, the endoscopic and pathological characteristics of colorectal MEITL were similar to those of ATLL.

A multicenter study from the Asia Lymphoma Study Group identified 38 MEITL patients, and the involved sites were the small intestine and stomach (5%), small intestine (63%), small and large intestine (16%), and large intestine (18%) [11]. MEITL can discontinuously expand into the mucosa along the entire GI tract. Aoyama et al. reported a primary colonic MEITL patient with Lugano clinical stage I, having mucosal edema and multiple ulcers in the transverse, splenic flexure, and sigmoid colon [24]. The current study also demonstrated that three MEITL patients (33%) had primarily presenting colorectal lesions. Endoscopically, six MEITL patients (67%) showed diffuse infiltrating type with more than two lymphomatous lesions in the whole colorectum. A previous study reported two colonic MEITL patients with clinicopathological features that mimicked those of ulcerative colitis and lymphocytic colitis, and the latter had a history of ‘lymphocytic colitis’ 6 months before [25]. Although collagenous sprue, microscopic (lymphocytic and collagenous) colitis, and celiac disease show distinct clinical entities, these diseases reveal similar features of colitis with an increase in T-IELs [26, 27]. Five examined MEITL patients (56%) had features of lymphocytic proctocolitis with reactive small T-IELs, and the findings were almost similar to lymphocytic colitis, in which more than 20 IELs per 100 epithelial cells and no or few distorted crypts were detected [28]. The features of lymphocytic proctocolitis may be characteristic of background mucosa in colorectal and other GI MEITL. Cumulative studies are necessary to clarify the relationship between lymphocytic proctocolitis and MEITL.

The prognosis of GI TNKCL patients is worse, and 1- and 3-year OS rates were 57 and 26%, respectively, in 42 intestinal MEITL patients [29]. Among them, OS of MEITL patients with Lugano clinical stages I, II1, and II2 was 18.8 months, which was significantly better than 4.9 months in stages IIE and IV (*P* = 0.01). The Lugano stage as well as the performance scales were important prognostic factors. Clinical stages in 55 GI EBV+ TNKCL patients and 61 GI-involved ATLL were also indicated as a significant prognostic factor (*P* < 0.001 and *P* = 0.017, respectively) [9, 23]. In the current study, only six of 27 colorectal TNKCL patients (22%) were found with stage I and II1 disease, and the 50% OS of the five was more than 240 months. The major symptom of examined colorectal TNKCL patients was chronic diarrhea. Thus, the clinical symptoms as well as the features of lymphocytic proctocolitis with CD8+ T-IELs may be important in detecting the early stages of colorectal TNKCL.

## Conclusions

MEITL, ATLL and other types of TNKCL occasionally involved the colorectal regions, and primary and concurrently involved colorectal MEITL was recognized. The endoscopic and clinicopathological characteristics of colorectal MEITL and ATLL revealed diffuse infiltrating lesions with increased atypical T-IELs. However, features of lymphocytic proctocolitis in the background mucosa were characteristic of colorectal MEITL. Primary colorectal MEITL patients in the early clinical stages were rarely identified and showed a rather prolonged clinical course. It is important to recognize endoscopic and clinicopathological characteristics including features of lymphocytic proctocolitis to detect early-stage colorectal TNKCL.

## Data Availability

The datasets used and analyzed in the current study are available from the corresponding author on reasonable request.

## Abbreviations

GI: Gastrointestinal
TNKCL: T/natural killer-cell lymphomas
EATL: Enteropathy-associated T-cell lymphoma
MEITL: Monomorphic epitheliotropic intestinal T-cell lymphoma
EBV: Epstein-barr virus
TLPD: T-cell lymphoproliferative disorder
ATLL: Adult T-cell leukemia/lymphoma
WHO: World health organization
IELs: Intraepithelial lymphocytes
OS: Overall survival
ALCL: Anaplastic large cell lymphoma
UC: Ulcerative colitis
LDH: Lactate dehydrogenase
sIL-2R: Soluble interleukin-2 receptor
